# p21 deficiency is susceptible to osteoarthritis through STAT3 phosphorylation

**DOI:** 10.1186/s13075-015-0828-6

**Published:** 2015-11-07

**Authors:** Shinya Hayashi, Takaaki Fujishiro, Shingo Hashimoto, Noriyuki Kanzaki, Nobuaki Chinzei, Shinsuke Kihara, Koji Takayama, Tomoyuki Matsumoto, Kotaro Nishida, Masahiro Kurosaka, Ryosuke Kuroda

**Affiliations:** Department of Orthopaedic Surgery, Kobe University Graduate School of Medicine, 7-5-1 Kusunoki-cho, Chuo-ku, Kobe, 650-0017 Japan

**Keywords:** p21, Chondrocytes, MMP-13, Aggrecan, STAT3

## Abstract

**Introduction:**

Osteoarthritis (OA) is a multifactorial disease, and recent studies have suggested that cell cycle–related proteins play a role in OA pathology. p21 was initially identified as a potent inhibitor of cell cycle progression. However, it has been proposed that p21 is a regulator of transcription factor activity. In this study, we evaluated the role of p21 in response to biomechanical stress.

**Methods:**

Human chondrocytes were treated with p21-specific small interfering RNA (siRNA), and cyclic tensile strain was introduced in the presence or absence of a signal transducer and activator of transcription 3 (STAT3)-specific inhibitor. Further, we developed an in vivo OA model in a p21-knockout background for in vivo experiments.

**Results:**

The expression of matrix metalloproteinase (*MMP13*) mRNA increased in response to cyclic tensile strain following transfection with p21 siRNA, whereas the expression of *aggrecan* was decreased. Phospho-STAT3 and MMP-13 protein levels increased following downregulation of p21, and this was reversed by treatment with a STAT3 inhibitor. p21-deficient mice were susceptible to OA, and this was associated with increased STAT3 phosphorylation, elevated MMP-13 expression, and elevation of synovial inflammation. The expression of p21 mRNA was decreased and phosphorylation of STAT3 was elevated in human OA chondrocytes.

**Conclusions:**

The lack of p21 has catabolic effects by regulation of aggrecan and MMP-13 expression through STAT3 phosphorylation in the cartilage tissue. p21 may function as a regulator of transcriptional factors other than the inhibitor of cell cycle progression in the cartilage tissue. Thus, the regulation of p21 may be a therapeutic strategy for the treatment of OA.

**Electronic supplementary material:**

The online version of this article (doi:10.1186/s13075-015-0828-6) contains supplementary material, which is available to authorized users.

## Introduction

The development of osteoarthritis (OA) is related to genetic factors, biomechanical stress, and biological factors within the joints [1–3]. Previous reports showed that mechanical stress can alter the matrix synthetic activity [4]. It has also been found that excessive mechanical stress may alter chondrocyte metabolism and reduce the amount of extracellular matrix (ECM) [5, 6]. Type II collagen and aggrecan (ACAN) are the main components of the articular cartilage ECM. In the OA cartilage environment, the loss of type II collagen and ACAN is due to decreased protein synthesis by chondrocytes and activation of ECM-degrading enzymes such as matrix metalloproteinases (MMPs) and a disintegrin and metalloproteinase with thrombospondin motifs (ADAMTS) [7]. The loss of ACAN through the activity of aggrecanase enzymes is a key event in early OA, and ADAMTS4 and ADAMTS5 are the major cartilage aggrecanases in humans [8]. MMPs, especially MMP-13, are critical enzymes involved in the degeneration of articular cartilage in OA [9].

The cyclin-dependent kinase inhibitor p21 was initially identified as a potent inhibitor of cell cycle progression [10–13]. Knockout of *p21* induced a regenerative response in an appendage of an otherwise nonregenerating mouse strain [14]. Subsequent studies further identified that p21 has an important role in controlling cytostasis and cell death [15]. *p21* transcription is activated by p53, and p21 is part of a negative feedback mechanism that controls p53 activity during apoptosis [16]. It is also an important mediator of inflammation and vascular smooth muscle cell proliferation [17]. Recently, p21 has been shown to regulate the activity of nuclear factor κB, c-Myc, CCAAT-enhancer-binding proteins, E2F, and signal transducer and activator of transcription 3 (STAT3) transcription factors [18–21]. p21 regulates cell proliferation and inflammation after arterial injury in local vascular cells [22]. Furthermore, downregulation of p21 increases the expression of stromal cell–derived factor 1 (SDF-1) via the STAT3 pathway [23]. A previous report showed that SDF-1 induced MMP-13 expression in chondrocytes [24]. Given these links between p21 and chondrocytes, a previous report showed that articular chondrocytes expressed high levels of p21 with positive senescent cells by senescence-associated β-galactosidase staining [25]. Another study showed that interleukin (IL)-1β increased proliferation and caused a G_1_-to-S phase shift in chondrocytes, accompanied by a reduction of *p21*, and reduction of *p21* caused delayed cell differentiation, manifested by decreases in proteoglycan synthesis, mineralization, alkaline phosphatase activity, and matrix proteins [26].

We initiated the present study to investigate the function of p21 in cartilage homeostasis. Specifically, we focused on the relationship between p21 and STAT3 in chondrocyte and joint biology in response to mechanical stress both in vitro and in vivo.

## Methods

### Preparation of human cartilage

Cartilage tissues were obtained during total hip joint replacement surgery from 11 patients with OA. Diagnosis of OA was based on clinical, laboratory, and radiographic evaluations. Normal cartilage tissues were obtained during surgery for femoral neck fractures from eight patients with no history of joint disease and with macroscopically normal cartilage. All the samples were obtained in accordance with the World Medical Association Declaration of Helsinki Ethical Principles for Medical Research Involving Human Subjects. The study protocol was approved by Kobe University Graduate school of Medicine Ethics Committee, and all patients gave their informed consent.

### Cell culture

Primary chondrocytes were isolated and cultured from the cartilage tissues. Tissues were minced and incubated with trypsin (0.5 mg/ml; Sigma-Aldrich, St. Louis, MO, USA) for 15 minutes at 37 °C, after which the cartilage was treated with Dulbecco’s modified Eagle’s medium (DMEM; Gibco/Life Technologies, Grand Island, NY, USA) containing 0.2 % collagenase (Sigma-Aldrich) at 37 °C for 15 h. Dissociated cells were cultured in DMEM supplemented with 10 % fetal bovine serum (BioWhittaker FBS; Lonza, Walkersville, MD, USA) and 100 U/ml penicillin-streptomycin. After overnight culture, nonadherent cells were removed and adherent cells were further incubated on a 6-well plate in fresh medium (3 × 10^5^ cells/well). All experiments were conducted using first-passage cells. To characterize the chondrocytes, we confirmed that the type II collagen showed higher expression in the normal human hip chondrocytes than in the OA knee chondrocytes and that type X collagen showed higher expression in the OA chondrocytes than in the normal chondrocytes.

### Cell culture and exposure to cyclic tensile strain

Normal human knee chondrocytes (NHAC-kn; Cambrex, Charles City, IA, USA) were cultured in a humidified atmosphere of 5 % CO_2_ and 95 % air at 37 °C in a BulletKit (Cambrex). NHAC-kn were derived from a single-donor knee articular cartilage and used as an established normal chondrocyte cell line [27, 28]. To characterize the chondrocytes, we confirmed that NHAC-kn expressed type II collagen and sulfated proteoglycans, but not type X collagen, before use.

Before we performed the experiments, cells were grown to a subconfluent state (3 × 10^5^ cells/well) in a 6-well plate and were then plated onto a silicon chamber coated with fibronectin (Sigma-Aldrich) at a density of 3 × 10^5^ cells/well in DMEM/F-12 supplemented with 10 % FBS and 100 U/ml penicillin-streptomycin. Cyclic tensile strain experiments were performed using an ST-140 cyclic tensile strain system (STREX, Osaka, Japan). Cyclic tensile strain was enforced at 3 %, 5 %, 8 %, and 10 % elongation for 3 h (0.5 Hz) according to previous studies [23, 29, 30]. We used 5 % tensile strain as normal joint loading and 10 % tensile strain as excessive loading to investigate the relationship between mechanical stress and cartilage metabolism.

### Small interfering RNA transfection

Lipofectamine 2000 reagent was used to transfect p21 small interfering RNA (siRNA) and nonspecific siRNA control into normal human knee chondrocyte monolayers according to the recommendations of the manufacturer (Life Technologies, Carlsbad, CA, USA). Briefly, 1 day before transfection, cells were plated in a 6-well plate in growth medium without antibiotics to attain 30–50 % confluence at the time of transfection. Subsequently, 100 pmol of siRNA and Lipofectamine 2000 complexes were prepared and added to each well. After 12 h of transfection, the complexes were removed, and fresh medium containing 10 % FBS was added. After an additional 12 h, the cells were replaced onto a silicone chamber coated with fibronectin at a density of 3 × 10^5^ cells/well, and cyclic tensile strain was applied.

### Quantitative reverse transcription polymerase chain reaction analysis

Chondrocytes were cultured in 6-well plates with various stimulations, and RNA was extracted using a QIAshredder and RNeasy Mini Kit (Qiagen, Hilden, Germany) according to the manufacturer’s protocol. Briefly, 1 μg of total RNA was reverse-transcribed to first-strand cDNA with 1.25 μM oligo(dT) primer in 40 μl of polymerase chain reaction (PCR) buffer II containing 2.5 mM MgC1_2_, 0.5 mM deoxyribonucleotide triphosphate mix, 0.5 U of RNase inhibitor, and 1.25 U of murine leukemia virus reverse transcriptase (PerkinElmer/Applied Biosystems, Foster City, CA, USA) at 42 °C for 60 minutes.

The relative expression levels of mRNA encoding human *p21*, collagen, type II, alpha 1 (*COL2A1*), *ACAN*, *MMP3*, *MMP13*, *ADAMTS4*, and *ADAMTS5* were analyzed by SYBR Green real-time PCR using an ABI Prism 7700 sequence detection system (Applied Biosystems). The relative expression of the genes of interest was normalized against the *GAPDH* housekeeping gene by using the comparative cycle threshold (C_t_) method. The difference between the mean C_t_ values of the gene of interest and the housekeeping gene is denoted as ΔC_t_, and the difference between ΔC_t_ and the C_t_ value of the calibrator sample is denoted as ΔΔC_t_. The log_2_ (ΔΔC_t_) value gives the relative level of gene expression. The primer sequence for detection of human *p21*, *COL2A1*, *ACAN*, *MMP3*, *MMP13*, *ADAMTS4*, and *ADAMTS5* are described in the Additional file 1.

### Western blot analysis

Chondrocytes were washed three times with phosphate-buffered saline and lysed in a hypotonic lysis buffer (25 mM Tris, 1 % Nonidet P-40, 150 mM NaCl, 1.5 mM ethylene glycol tetraacetic acid) supplemented with a protease and phosphatase inhibitor mix (Roche Diagnostics, Basel, Switzerland) on ice for 20 minutes [31]. The lysates were centrifuged at 15,000 rpm for 20 minutes to remove cellular debris, and the supernatants were collected. Cytoplasmic proteins were quantified by using the Bradford method with a protein assay reagent (Bio-Rad Laboratories, Hercules, CA, USA) and diluted to an equal concentration with the hypotonic buffer. The expression of p21 protein was detected using rabbit anti-p21 polyclonal antibody (Ab) (Cell Signaling Technology, Danvers, MA, USA). The expression levels of phosphorylated STAT3 (phospho-STAT3 or p-STAT3) and STAT3 were detected using rabbit anti-phospho-STAT3 and anti-STAT3 Ab (Cell Signaling Technology).

Horseradish peroxidase (HRP)-conjugated goat anti-rabbit immunoglobulin G (IgG) Ab (GE Healthcare Bio-Sciences, Piscataway, NJ, USA) or HRP-conjugated rabbit anti-mouse IgG Ab (GE Healthcare Bio-Sciences) was used as a secondary antibody, and the signals were visualized using Amersham ECL Plus reagent (GE Healthcare Life Sciences, Little Chalfont, UK) with the LAS-3000mini chemiluminescent image analyzer (FUJIFILM Life Science, Tokyo, Japan). Actin was used as a control to estimate protein loading on the gel.

### Generation of homozygous mice

Homozygous B6.129S6(Cg)-*Cdkn1a*^*tm1Led*^/J mice were obtained from The Jackson Laboratory (Bar Harbor, ME, USA). We backcrossed the mice against a C57BL/6 background and studied male mice at 10 weeks of age. All the mice used in this study were backcrossed over 20 generations. *p21*^*+/+*^ littermates were used as wild-type controls. Genotyping was performed by PCR amplification of mouse-tail DNA by using allele-specific probes. Each experimental group contained at least six mice.

### In vivo mouse OA model

This study was carried out in strict accordance with the recommendations contained in the Guide for the Care and Use of Laboratory Animals of the National Institutes of Health. All procedures were approved by the Animal Studies Committee of Kobe University, Japan (permit number P131104). Ten-week-old male p21^−/−^ and p21^+/+^ mice were used in these experiments. Mice were anesthetized using an intraperitoneal injection of ketamine (100 mg/kg). Destabilization of the medial meniscus (DMM) was induced in the right knee joint by transecting the anterior attachment of the medial meniscotibial ligament as described previously [32]. For the control, surgery was performed on the right knee joints where the ligaments were intact and termed *sham*. All mice were subjected to weight-bearing following recovery from anesthesia. The mice were sacrificed 8 weeks after DMM or sham surgery, and their tissue was subjected to histological evaluation. Six mice were analyzed for each group: p21^+/+^ sham, p21^+/+^ DMM, p21^−/−^ sham, and p21^−/−^ DMM (total of 24 analyzed mice).

### Histological evaluation for cartilage degeneration

Mouse knee joints were fixed with 4 % paraformaldehyde for 24 h, decalcified with 14 % ethylenediaminetetraacetic acid for 7 days, and embedded in paraffin. Coronal histological sections were taken through the joint at 80-μm intervals and stained with Safranin O and Fast Green. OA histopathology was evaluated by using the Osteoarthritis Research Society International (OARSI) cartilage OA histopathology scoring system [33]. Histological scores were measured in four quadrants (medial femoral condyle, medial tibial plateau, lateral femoral condyle, and lateral tibial plateau) of the knee joints at all sectioned levels (eight sections per knee). A summed OA score was calculated from all four quadrants for all sections, which represented the changes across the whole joint.

### Immunohistochemistry

Deparaffinized sections were digested with proteinase (Dako, Glostrup, Denmark) for 10 minutes and treated with 3 % hydrogen peroxide (Wako Pure Chemical Industries, Osaka, Japan) to block endogenous peroxidase activity. The sections were treated with a 1:100 dilution of antiphosphorylated STAT3 (Cell Signaling Technology), anti-MMP-13 (Abcam, Cambridge, UK), and anti-F4/80 (AbD Serotec, Kidlington, UK) antibodies at 4 °C overnight and were subsequently treated with peroxidase-labeled antirabbit Ig (Histofine Simple Stain MAX PO; Nichirei Bioscience, Tokyo, Japan) at room temperature for 30 minutes. The signal was developed as a brown reaction product by using the peroxidase substrate 3,3′-diaminobenzidine (Histofine Simple Stain DAB Solution; Nichirei Bioscience), and the sections were examined microscopically. Methyl green stain was used as a counterstain. One sagittal section from the center of the most severe OA lesion in each tibial plateau was scored. The number of stained cells was counted in three areas of high-magnification fields at both superficial and deep zones of the cartilage tissue individually by three blinded observers. The average percentage of p-STAT3– and MMP-13–positive cells/total cells were calculated. One sagittal section each from six mice was evaluated in each group. These positive cells were included superior to the tidemark and were included for both femur and tibia.

### Statistical analysis

**Fig. 1:**
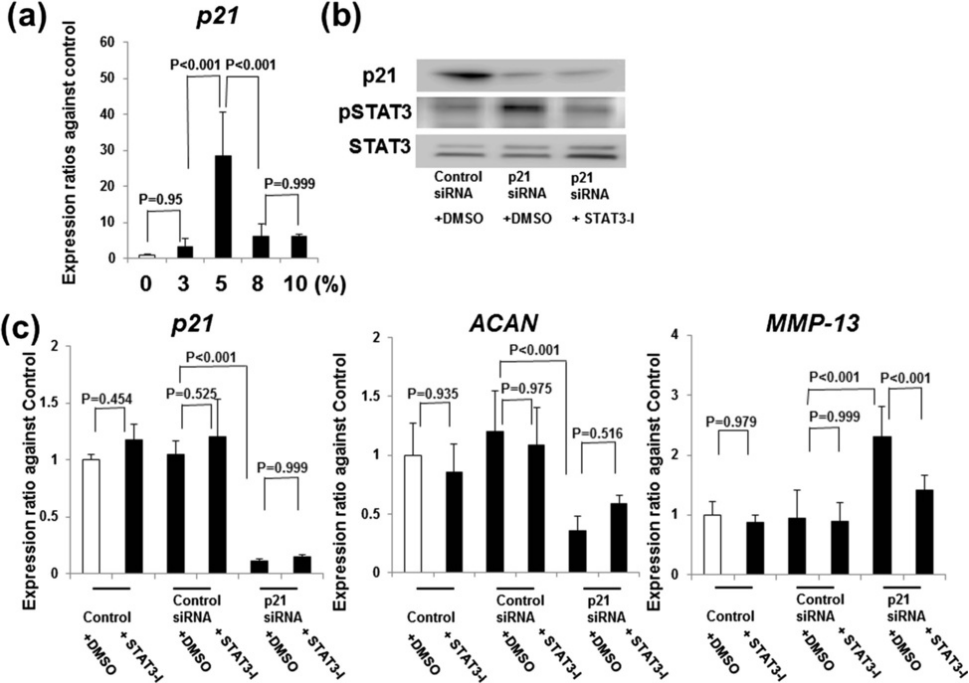
Expression of p21, ACAN, and MMP-13 without and with STAT3 inhibitor. **a** Expression levels *of p21* mRNA were quantified by real-time PCR. Expression ratios of *p21* are shown. Columns represent mean ratios with 95 % CI at 3, 5, 8, and 10 % strain/nonstrain control. **b** p21 and phospho-STAT3 were analyzed by Western blotting. The results shown represent three independent experiments. **c** The effect of a STAT3-specific inhibitor on p21-regulated ACAN and MMP-13 expression. Chondrocytes were transfected with p21 siRNA or nonspecific control siRNA for 12 h, and 5 % cyclic stretch stress was introduced for 3 h in the presence of 50 μM DMSO or STAT3-specific inhibitor. Expression levels of *p21*, *ACAN*, and *MMP13* mRNAs were quantified by real-time PCR. Expression ratios of *ACAN* and *MMP13* are shown. Columns represent mean ratios with 95 % CI against untreated control. The results shown are the average of four individual experiments. Values are normalized to *GAPDH* expression. *ACAN* aggrecan, *CI* confidence interval, *DMSO* dimethyl sulfoxide, *MMP* matrix metalloproteinase, *PCR* polymerase chain reaction, *siRNA* small interfering RNA, *STAT3-I* signal transducer and activator of transcription 3–specific inhibitor

**Fig. 2:**
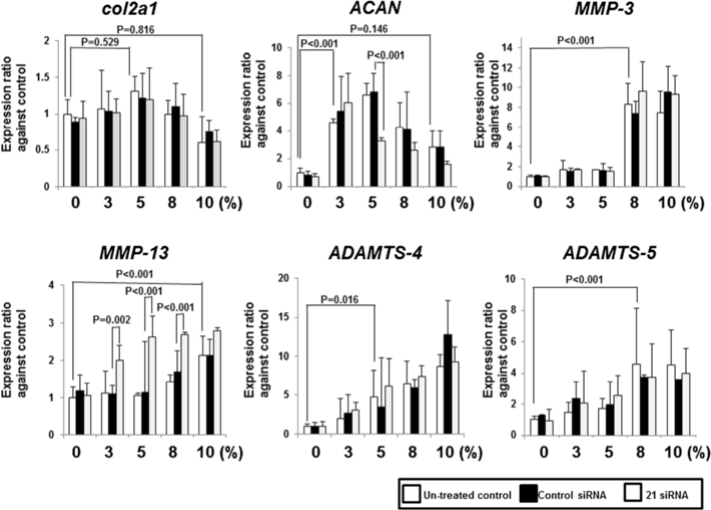
Knockdown efficiency of p21 siRNA transfection in human normal chondrocytes in response to mechanical strain. The concentration of p21 siRNA was 100 nM. Expression levels of *p21*, *COL2A1*, *ACAN*, *MMP3*, *MMP13*, *ADAMTS4*, and *ADAMTS5* mRNAs were quantified by real-time PCR. Values are normalized to *GAPDH* expression. Columns represent mean ratios with 95 % confidence intervals against untreated and nonloaded control. The results shown are the average of four individual experiments. White columns: untreated samples, Black columns: nonspecific control siRNA samples, and gray columns: p21 siRNA samples. *ACAN* aggrecan, *ADAMTS* a disintegrin and metalloproteinase with thrombospondin motifs, *COL2A1* collagen, type II, alpha 1, *MMP* matrix metalloproteinase; *PCR* polymerase chain reaction, *siRNA* small interfering RNA

**Fig. 3:**
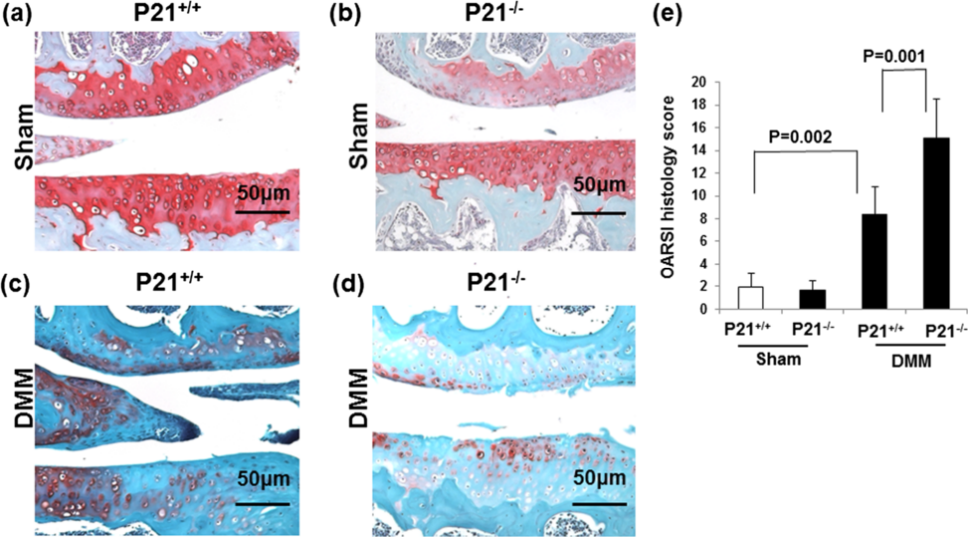
p21 levels influence the summed OARSI scores in DMM mice. Cartilage tissue samples were collected from the right knee (sham or DMM surgery) of (**a**) p21^+/+^ (sham), (**b**) p21^−/−^ (sham), (**c**) p21^+/+^ (DMM), and (**d**) p21^−/−^ (DMM) mice. The sections are stained with Safranin O and Fast Green. **e** Average summed OARSI scores with 95 % CIs from all four quadrants and eight sections. Six mice were analyzed for each group: p21^+/+^ sham, p21^+/+^ DMM, p21^−/−^ sham, and p21^−/−^ DMM. *CI* confidence interval, *DMM* destabilization of the medial meniscus, *OARSI* Osteoarthritis Research Society International

**Fig. 4:**
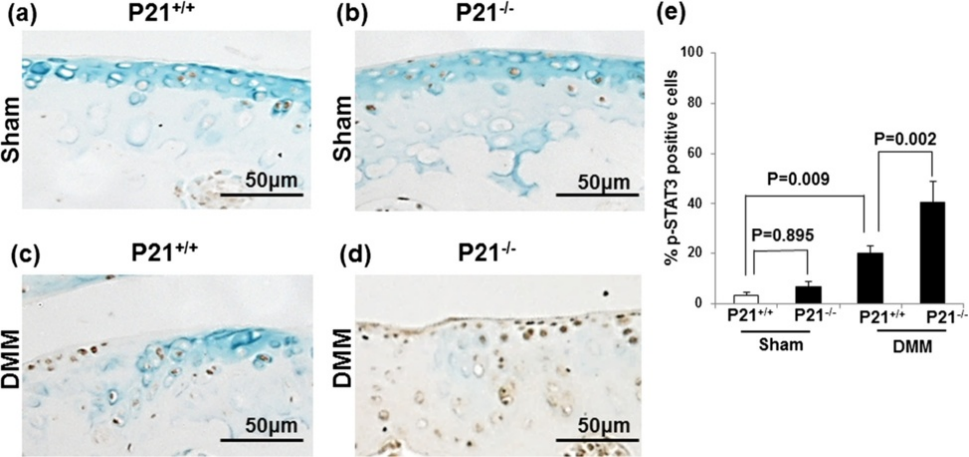
p21 levels influence the number of p-STAT3-positive cells in DMM mice. Cartilage tissue samples were collected from the right knee (sham or DMM surgery) of (**a**) p21^+/+^ (sham), (**b**) p21^−/−^ (sham), (**c**) p21^+/+^ (DMM), and (**d**) p21^−/−^ (DMM) mice. **e** Percentages of pSTAT3-positive stained cells (number of positive cells/number of total cells) with 95 % CI. The sections are stained for p-STAT3 antibody and counterstained with methyl green. Six mice were analyzed for each of the following groups: p21^+/+^ sham, p21^+/+^ DMM, p21^−/−^ sham, and p21^−/−^ DMM. *CI* confidence interval, *DMM* destabilization of the medial meniscus, *p-STAT3* phosphorylated signal transducer and activator of transcription 3

**Fig. 5:**
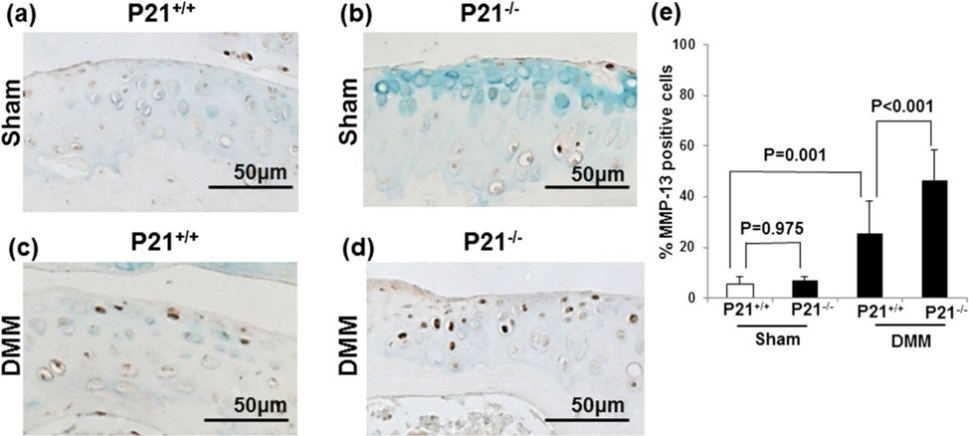
p21 levels influence the number of MMP-13–positive cells in DMM mice. Cartilage tissue samples were collected from the right knee (sham or DMM surgery) of (**a**) p21^+/+^ (sham), (**b**) p21^−/−^ (sham), (**c**) p21^+/+^ (DMM), and (**d**) p21^−/−^ (DMM) mice. **e** Percentages of MMP-13–positive stained cells (number of positive cells/number of total cells) with 95 % CI. The sections are stained for MMP-13 antibody and counterstained with methyl green. Six mice were analyzed for each group: p21^+/+^ sham, p21^+/+^ DMM, p21^−/−^ sham, and p21^−/−^ DMM. *CI* confidence interval, *DMM* destabilization of the medial meniscus, *MMP* matrix metalloproteinase

**Fig. 6:**
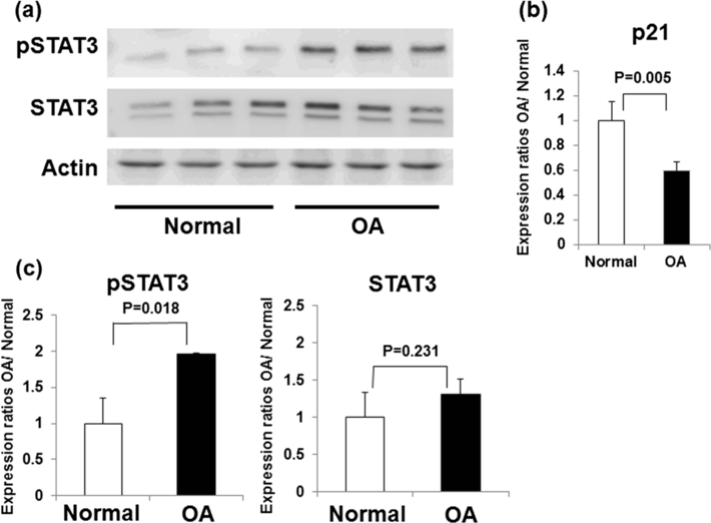
Differential expression of p21, STAT3, and p-STAT3 in normal and OA chondrocytes. **a** Expression of p-STAT3 and total STAT3 in normal (three samples) and OA (three samples) primary chondrocytes analyzed by Western blotting. **b** Columns represent the ratios of protein expression levels (p-STAT3/actin, STAT3/actin) determined by semiquantification of the digitally captured image. The results shown are the averages of three individual samples. **c** Expression levels of p21 mRNA levels were quantified by real-time PCR. Expression ratios of p21 are shown. Columns represent mean ratios with 95 % CIs of primary OA chondrocytes/primary normal chondrocytes. The results shown are the averages of five individual samples. Values are normalized to *GAPDH* expression. *CI* confidence interval, *OA* osteoarthritis, *p-STAT3* phosphorylated signal transducer and activator of transcription 3, *STAT3* signal transducer and activator of transcription 3

Statistical analysis was performed using one-way (Fig. 1a, c) or two-way (Figs. 2, 3e, 4e and 5e) analysis of variance with Tukey’s post hoc test for multiple comparisons of paired samples. The Mann–Whitney *U* test was used for comparisons between the two groups (Fig. 6a, b). *P* values less than 0.05 were considered significant. The results are presented as mean values with 95 % confidence intervals (95 % CIs) and were considered statistically significant at *P* < 0.05.

## Results

### Cyclic tensile strain increased catabolic genes

Real-time PCR analysis showed that the expression of *Col2a1* in untreated control mice did not change in response to tensile strain. *ACAN* in untreated control mice increased after 3 %, 5 %, and 8 % strain; however, the expression levels were decreased after 10 % strain. Moreover, *MMP3* in untreated control mice was increased after 8 % and 10 % strain, and *MMP13* was increased after 10 % strain. *ADAMTS4* in untreated control mice was increased after 5 %, 8 %, and 10 % strain, and *ADAMTS5* was increased after 8 % and 10 % strain (Fig. 2). These results indicate that cyclic tensile strain increased the expression levels of ACAN and catabolic genes.

### Downregulation of p21 expression decreased aggrecan expression and increased MMP-13 expression

To analyze the function of p21 in chondrocytes in response to mechanical stress, we compared *COL2A1*, *ACAN*, *MMPs*, and *ADAMTS* mRNA levels in p21-knockdown chondrocytes subjected to cyclic tensile strain.

Real-time PCR analysis showed that *p21* expression was inhibited by p21-specific siRNA transfection in all cells subjected to tensile strain (Fig. 2). Downregulation of *p21* led to a reduction in *ACAN* expression after 5 % strain in comparison with control siRNA samples, and it increased *MMP13* expression after 3 %, 5 %, and 8 % strain (Fig. 2). However, the expression levels of *ACAN and MMP13* were unchanged in unloaded samples (Fig. 2).

### Cyclic tensile strain increased p21 mRNA expression

To evaluate the expression levels of *p21*, we compared the *p21* levels in chondrocytes subjected to cyclic tensile strain. Real-time PCR analysis showed that the expression levels of *p21* increased after 5 % strain (Fig. 1a).

### Inhibition of STAT3 activation blocks the p21-dependent regulation of MMP13 but not aggrecan expression

To understand the mechanism by which p21 levels affect *ACAN* and *MMP13* expression, we pretreated the control and p21-knockdown cells with a STAT3 inhibitor and measured gene expression after application of 5 % cyclic tensile strain.

Western blot analysis confirmed that the phosphorylation of STAT3 increased after p21 knockdown, but the effect was abolished with a STAT3 inhibitor (Fig. 1b). Real-time PCR analysis showed that STAT3 inhibitor itself did not affect *p21*, *ACAN*, or *MMP13* expression (Fig. 1c). *MMP13* expression decreased upon STAT3 inhibition after strain application, but *ACAN* levels were unchanged (Fig. 1c).

### p21 deficiency is susceptible to OA change in vivo

To determine the effect of p21 in vivo, we developed a DMM model in p21-knockout mice and compared these animals with wild-type mice 8 weeks after surgery. Safranin O and Fast Green staining revealed that wild-type mice showed retention of the articular surface layer; however, loss of Safranin O staining was observed (Fig. 3c). p21-knockout mice showed midzone excavation of the cartilage tissue and loss of hyaline cartilage proteoglycan staining (Fig. 3d). Wild-type or p21-knockout mice did not show matrix depression in the sham surgery control group (Fig. 3a, b). According to the OARSI cartilage OA histopathology scoring system, the average sum score was significantly increased in the DMM wild-type mice compared with sham wild-type mice. Further, the average sum score was significantly higher in the DMM p21-knockout mice than in the DMM wild-type mice (Fig. 3e).

### p21 deficiency is susceptible to OA change through p-STAT3 phosphorylation and MMP-13 expression

Immunohistochemistry showed that the expression levels of both p-STAT3 and MMP-13 in the DMM wild-type mice were elevated in comparison with the sham wild-type mice (Figs. 4a, c and 5a, c). Further, the expression levels of p-STAT3 and MMP-13 were elevated in the DMM wild-type and p21-knockout mice (Figs. 4c, d and 5c, d). The percentage of p-STAT3– and MMP-13–positive cells were significantly increased in the DMM p21-knockout mice compared with the DMM wild-type mice (Figs. 4e and 5e). These results indicate that p21 deficiency affects the phosphorylation of STAT3 and MMP-13 expression and confirm our in vitro experiments.

### p21 deficiency increased p-STAT3 phosphorylation and F4/80 expression in the synovial tissue

**Fig. 7:**
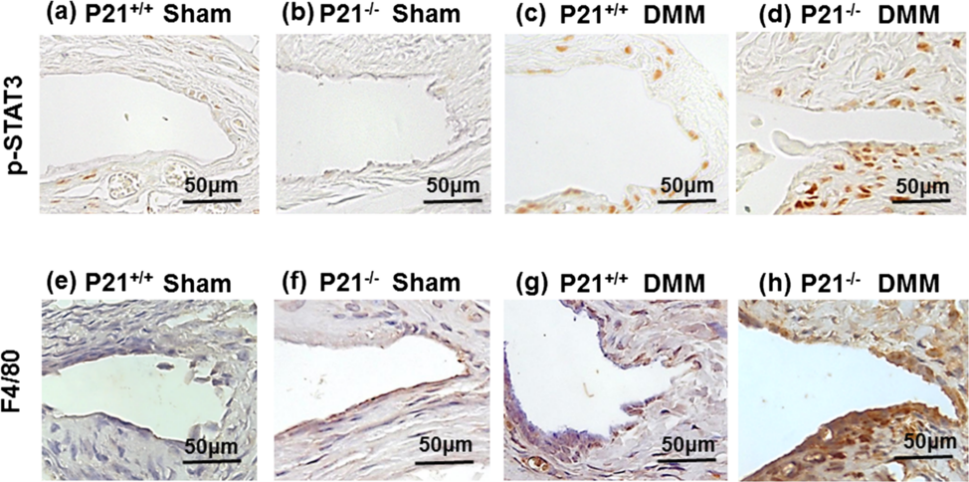
p21 levels influence p-STAT3 and F4/80 levels in DMM mice. Synovial tissue samples were collected from the right knee (sham or DMM surgery) of (**a**, **e**) p21^+/+^ (sham), (**b**, **f**) p21^−/−^ (sham), (**c**, **g**) p21^+/+^ (DMM), and (**d**, **h**) p21^−/−^ (DMM) mice. The sections are stained for p-STAT3 Ab (*upper panels*) and F4/80 Ab (*lower panels*) and counterstained with methyl green. *Ab* antibody, *DMM* destabilization of the medial meniscus, *p-STAT3* phosphorylated signal transducer and activator of transcription 3

Immunohistochemistry of the synovial tissue showed that the expression levels of p-STAT3 in the DMM wild-type mice were elevated in comparison with the sham wild-type and p21-knockout mice (Fig. 7a–c), but the expression levels in the DMM p21-knockout mice were substantially elevated in comparison with the DMM wild-type mice (Fig. 7c, d). To determine whether p21 is implicated in the inflammatory response of the synovial tissue in experimental OA models, we investigated F4/80 expression as an immune and inflammatory cell marker because it is a well-known macrophage marker. Expression levels of F4/80 were similar to p-STAT expression. The expression levels in the DMM p21-knockout mice were elevated much higher in comparison with the DMM wild-type mice (Fig. 7e–h). These results indicate that p21 deficiency impacts phosphorylation of STAT3 and susceptibility to synovial inflammation.

### Phosphorylation of STAT3 was increased in human OA chondrocytes

We compared the phosphorylation of STAT3 in OA and normal primary chondrocytes. Western blot analysis showed that the expression levels of STAT3 were not significantly changed in normal primary chondrocytes. However, the phosphorylation of STAT3 was significantly increased in OA chondrocytes (Fig. 6a).

### Expression level of p21 was decreased in human OA chondrocytes

To analyze the expression levels of p21 and cartilage matrix–related genes in human chondrocytes, we compared p21 mRNA levels between human normal and OA chondrocytes. Real-time PCR showed that the p21 mRNA levels in OA chondrocytes were significantly decreased in comparison with the normal chondrocytes (Fig. 6b).

## Discussion

We first demonstrated that the downregulation of p21 decreased *ACAN* expression and increased *MMP13* expression following strain application. However, the downregulation of p21 did not change *ACAN* or *MMP13* expression in control cells not subjected to strain. We concluded that p21 functioned only under mechanical stress. We also demonstrated that the downregulation of p21 did not increase MMP13 expression after 10 % strain. *MMP13* expression levels in untreated siRNA chondrocytes were increased after 10 % strain. Therefore, the effect of p21 knockdown on *MMP13* expression may be attenuated by the upregulated MMP-13 expression. Further, downregulation of p21 did not change *ACAN* expression in response to 3 % and 10 % strain. The expression levels of p21 under 3 % and 10 % were much lower than 5 %, and the results may be dependent on p21 expression levels.

O’Connor reported that the maximum principal stress, though compressive under the region of contact, becomes tensile outside the contact patch, and localized tensile stress occurs in regions close to the cartilage–bone interface as well as at the articular surface [34] . In physiological circumstances, articular chondrocytes are exposed to a 15 % compression load, leading to 5 % tensile strain on chondrocytes during normal joint movement [35]. Therefore, we used 5 % tensile strain as normal joint loading and 10 % tensile strain as excessive loading to investigate the relationship between mechanical stress and cartilage metabolism.

To determine the mechanism underlying the changes in *ACAN* and *MMP13* expression, we focused on the Janus kinase (JAK)-STAT signaling pathway. Recently, it was reported that the JAK-STAT signaling pathway plays a critical role in *MMP13* expression in chondrocytes [36]. Legendre et al. demonstrated that the inhibition of the JAK-STAT pathway with a specific inhibitor diminished IL-6–induced *MMP13* expression in chondrocytes [37]. These results support our findings that the reduction of p21 increases STAT3 phosphorylation and that inhibition of STAT3 phosphorylation diminishes p21-regulated MMP-13 expression after mechanical stress. However, the inhibition of STAT3 phosphorylation did not diminish p21-regulated *ACAN* expression. Therefore, p21-regulated *ACAN* expression does not appear to be controlled by STAT3 signaling. Dai et al. demonstrated that the catabolic stress induced by IL-1β or hydrogen peroxide increased caveolin 1 and p21 expression in chondrocytes and that overexpression of caveolin 1 increased p21 expression and p38 mitogen-activated protein kinase activation, as well as impaired the ability of chondrocytes to induce type II collagen and ACAN expression [38]. Those authors concluded that p21 is a negative regulator of type II collagen and ACAN synthesis. However, a direct demonstration that the p21 levels control the expression of type II collagen and ACAN was not performed in the aforementioned study. In contrast to the results of that study [35], we demonstrated that a reduction of p21 expression decreased ACAN expression after mechanical stress.

We established a mouse OA model and examined the role of p21-knockout in this setting. We showed that p21-deficient mice were susceptible to OA and this was associated with increased STAT3 phosphorylation and MMP-13 expression. These results supported our in vitro experiments.

Lee et al. reported that IL-17 increased the expression of Toll-like receptor 3 via the STAT3 pathway in rheumatoid arthritis (RA) fibroblast-like synoviocytes and that STAT3 phosphorylation was increased in the synovial samples from patients with RA compared with patients with OA [39]. However, there was no report about STAT3 phosphorylation in comparison with OA and normal cartilage. We first demonstrated that STAT3 phosphorylation was elevated in OA compared with normal human cartilage and mouse OA model cartilage. We further demonstrated that the expression levels of p21 were decreased in human OA chondrocytes. Our findings support the following hypothesis for the regulation of MMP-13 expression by p21 under physiological conditions: p21 expression is decreased in the OA joint, leading to an increase in STAT3 phosphorylation and *MMP13* expression in chondrocytes.

Macrophages play a critical role in inflammation with three major functions—antigen presentation, phagocytosis, and immunomodulation—through the production of various cytokines and growth factors [40]. Recently, Mavers et al. demonstrated that enhanced and sustained development of experimental inflammatory arthritis, associated with markedly increased number of macrophages and severe articular destruction, was observed in p21-knockout mice [41]. Moreover, several authors have reported the interaction between inflammation and OA development [42–44] . OA is a much more complex disease, with inflammatory mediators released by cartilage, bone, and synovium [42]. We also confirmed the susceptibility to synovial inflammation in p21-knockout mice by DMM surgery. Therefore, the susceptibility to inflammation in p21-knockout mice may be one of the reasons that the p21-knockout mouse could easily develop OA by DMM surgery.

## Conclusions

The lack of p21 has catabolic effects by regulation of ACAN and MMP-13 expression through STAT3 phosphorylation in the cartilage tissue. p21 may function as a regulator of transcription factors in addition to being an inhibitor of cell cycle progression in the cartilage tissue. Thus, the regulation of p21 may be a therapeutic strategy for the treatment of OA. However, p21 also plays a role as a cell cycle regulator and as an oncogene. Therefore, further work is required to verify that the stabilization of p21 would be a viable and safe strategy for OA treatment.

## Additional file

Additional file 1: The primer sequence for detection of human p21, COL2A1, ACAN, MMP-3, MMP-13, ADAMTS-4, ADAMTS-5. (TIFF 58 kb)

## Abbreviations

Ab: Antibody
ACAN: Aggrecan
ADAMTS: A disintegrin and metalloproteinase with thrombospondin motifs
CI: Confidence interval
COL2A1: Collagen, type II, alpha 1
C_t_: Cycle threshold
DAB: 3,3′-diaminobenzidine
DMEM: Dulbecco’s modified Eagle’s medium
DMM: Destabilization of the medial meniscus
DMSO: Dimethyl sulfoxide
ECM: Extracellular matrix
FBS: Fetal bovine serum
HRP: Horseradish peroxidase
Ig: Immunoglobulin
IL: Interleukin
JAK: Janus kinase
MMP: Matrix metalloproteinase
NHAC-kn: Normal human knee chondrocytes
OA: Osteoarthritis
OARSI: Osteoarthritis Research Society International
PCR: Polymerase chain reaction
phospho-STAT3 or p-STAT3: Phosphorylated signal transducer and activator of transcription 3
RA: Rheumatoid arthritis
SDF-1: Stromal cell-derived factor 1
siRNA: Small interfering RNA
STAT3: Signal transducer and activator of transcription 3
STAT3-I: Signal transducer and activator of transcription 3–specific inhibitor
