# Supplementary figures and images for: p21 deficiency is susceptible to osteoarthritis through STAT3 phosphorylation
Source: Arthritis Res Ther. 2015 Nov 7;17:314. doi: 10.1186/s13075-015-0828-6 (PMC4636813; doi:10.1186/s13075-015-0828-6)

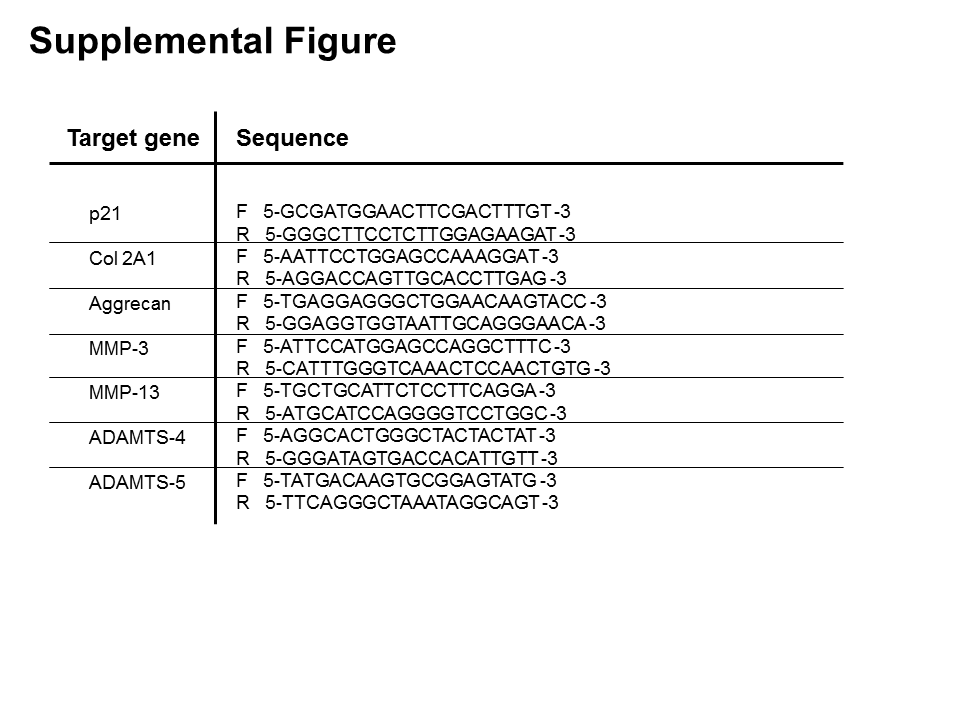

Supplement: Additional file 1: — The primer sequence for detection of human p21, COL2A1, ACAN, MMP-3, MMP-13, ADAMTS-4, ADAMTS-5. (TIFF 58 kb) [file 13075_2015_828_MOESM1_ESM.tiff]
